# The use of an omental flap for the reconstruction of a burn injury to the scalp: A case report

**DOI:** 10.1016/j.ijscr.2018.11.038

**Published:** 2018-11-22

**Authors:** Noor H. AlLababidi, Asal AlOmran, Fuad K. Hashem

**Affiliations:** a College of Medicine, Imam Abdulrahman Bin Fasial University, Dammam, Saudi Arabia; b College of Medicine, AlFaisal University, Riyadh, Saudi Arabia; c King Faisal Specialist Hospital and Research Center (KFSHRC), Consultant Plastic Surgery, Riyadh, Saudi Arabia

**Keywords:** Head and neck, Scalp, Reconstruction, Burn injury, Plastic surgery, Omental flap

## Abstract

**Introduction:**

Omental free flaps are used in reconstructing a wide range of clinical indications in the head and neck. The first omental flap case to be reported was in 1972 by McLean and Buncke.

**Case presentation:**

This case demonstrate the feasibility of minimal invasive procedure of harvest an omental flap laparoscopically to cover the entire scalp after a burn injury in a young male. It is first to be reported in Saudi Arabia at our institute, Riyadh, 2012. A 14-year-old male had an extensive flame burn at the age of 2 years in the head and neck area. He received multiple skin grafting procedures and an amputation of the right hand, due to his chronic condition of recurrent open wounds the patient was admitted for removal of the unstable ulcer sting and scarred skin of the scalp and for coverage with omental free flap and split thickness skin graft.

**Discussion:**

The coverage of wounds in the head and neck is usually done by using a local skin flap. The disadvantage of using the traditional skin flaps is difficulty to shape it in irregular, and large wounds.

**Conclusion:**

Owing its large size in covering wide defect, easy to shape in irregular edges over the bony surfaces, and high vascularity. This case demonstrate the feasibility of minimal invasive procedure of harvest an omental flap laparoscopically. We think that the use of omental flap is a useful flap and should be considered in the scalp and head and neck reconstruction.

## Highlights

• Good out come with few adverse effect of a minimal invasive procedure.
• Renewal of a known method in head and neck reconstruction.
• The first reported reconstruction due to flame burn injury.

## 1 Introduction

Omental free flaps are used in reconstructing a wide range of clinical indications in the head and neck, including post tumor resection and burns in the scalp, craniofacial reconstruction after tumor resection, hemi facial atrophy, trauma, and after tumor resection and radiation in the neck [1].

The first reported omental flap case was in 1972 by McLean and Buncke [1] when a free omental transplant by microsurgical anastomosis was performed to cover a scalp defect after a right temporoparietal neurofibroma was removed. The introduction of the omental flap procedure was successful and is now used in various reconstructive surgeries [2].

The omental free flap has great advantages due to its good vascularity, large size, and feasibility to shape. It also provides minimal donor site morbidity. [3]

This case demonstrate the feasibility of minimal invasive procedure of harvest an omental flap laparoscopically, and the use of large omental flap to cover the entire scalp after a burn injury of a child skull the probably even latissimus dorsi flap may not provide similar coverage and pliability.

It is the first to be reported in Saudi Arabia, and it was done at our institute, Riyadh, 2012.

The case has been reported in line with the SCARE criteria [7]. Parental consent on behalf of the patient has been obtained for publication of this case report.

## 2 Case presentation

A 14-year-old male had an extensive flame burn in the right side of neck, face, and scalp, anterior chest, and right upper limb at the age of 2 years.

No past medical or surgical history. No known drug history, no family history of any genetic disorder, patient and both parents are non-smokers.

He received multiple skin grafting procedures and an amputation of the right hand. The burn to his scalp was treated with a burr hole to the outer cortex to allow granulation tissue to cover the scalp, and then the wound was skin grafted.

The patient chronically continued to have unstable skin over the scalp and kept developing recurrent ulceration. On 29 March 2012, the patient was admitted throw the clinic due to his chronic condition of recurrent open wounds for many years.

**Fig. 1:**
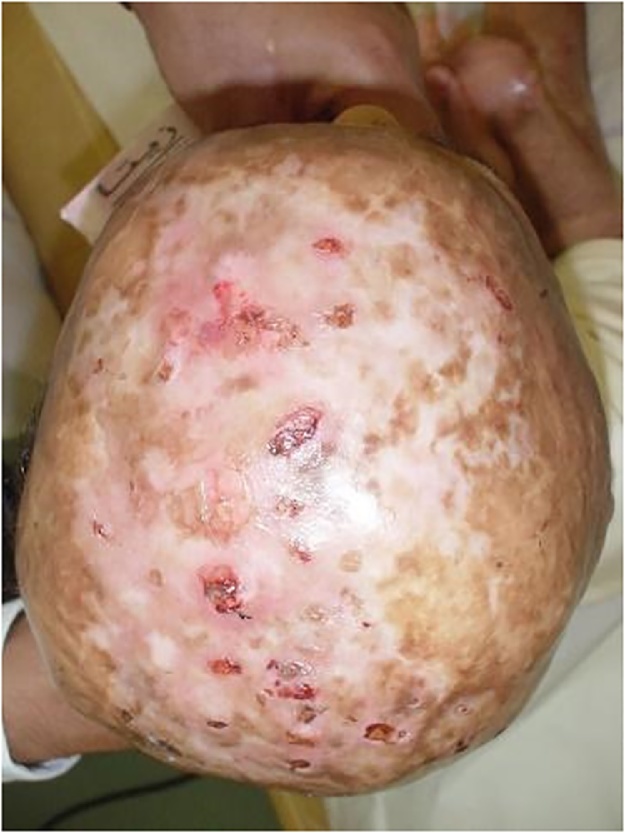
pre-operative unstable skin over the scalp.

A plan was set for the removal of the unstable ulcer and scarred skin of the scalp and coverage with an omental free flap and split thickness skin graft (Fig. 1).

Under general anesthesia with endotracheal intubation, the patient was in supine position, with draping of the head circumflex, face, abdomen, and left thigh down to the knee.

**Fig. 2:**
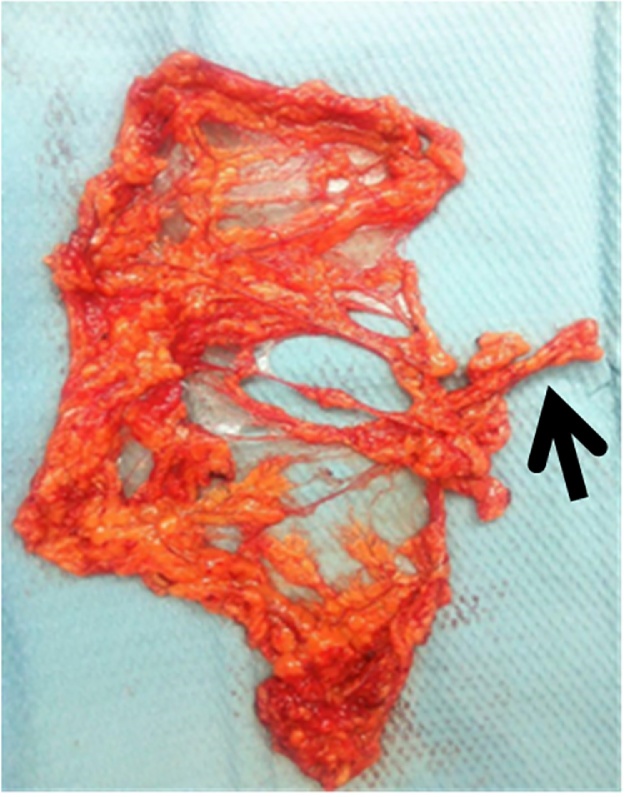
free omental flap with the Left gastroepiploics artery.

The General Surgery team was consulted for laparoscopically harvesting the omental flap and was performed by Dr I.Anwar based on the left gastroepiploic artery. The right superficial temporal artery was identified to be the recipient vessel (Fig. 2).

The ENT team did a right neck exploration to explore the neck vessels as a backup in case the right superficial temporal artery was not suitable.

The free omental flap based on the left gastroepiploic artery was anastomosed to the right superficial temporal artery. The micro procedure of omental free flap was performed by DR F.Hashem, Plastic surgeon.

The patient was given heparin 2000 U intraoperatively. The flap was viable with bleeding with a positive Doppler signal.

**Fig. 3:**
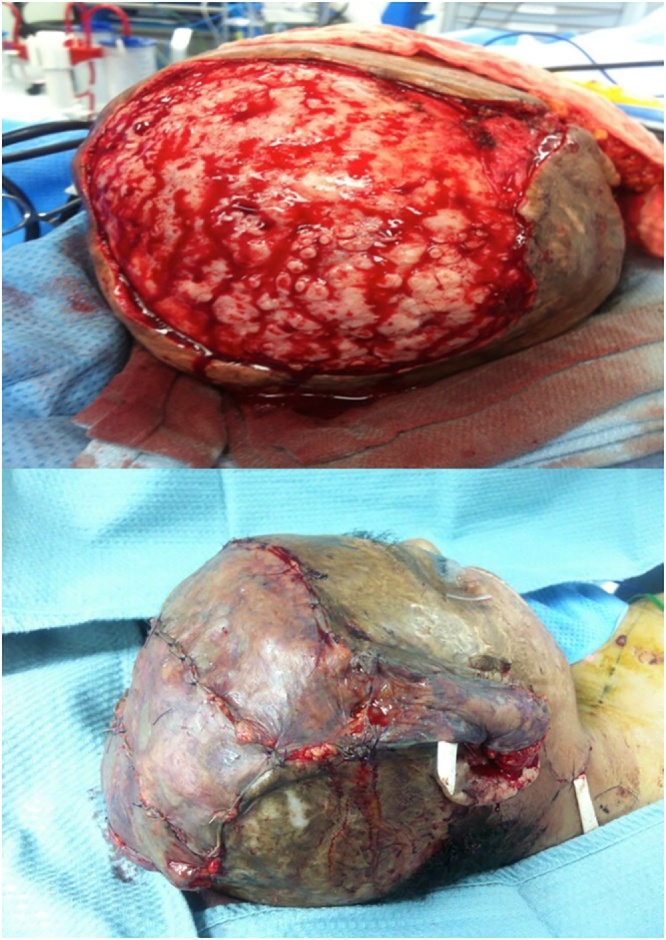
Flap placement.

After establishing flap revascularization, all the excising unstable scarred and ulcerated skin covering the scalp was resected, and the underlying irregular skull was smoothed down using a flat burr. The omental flap then covered the scalp, and a split-thickness skin graft from the thigh was placed over the omental flap (Fig. 3).

The patient was monitored postoperatively in intensive care unit. Post-operative care for microsurgery and wound care for skin graft over omental flap.

The hospital course was uneventful. The patient was kept on IV anticoagulants (dextran and heparin) for 5 days.

**Fig. 4:**
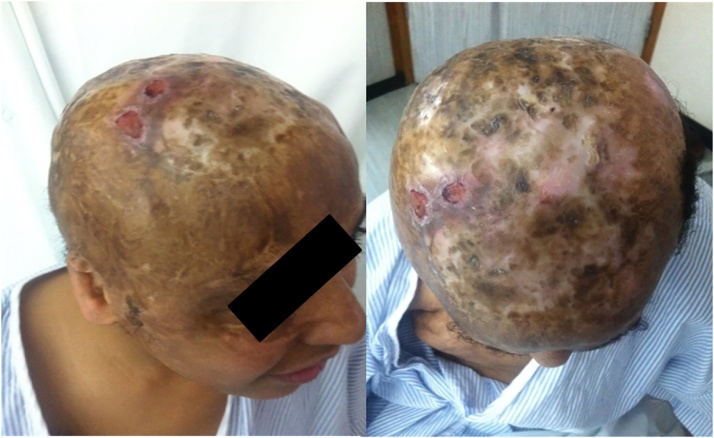
post-operative result.

The patient tolerated the procedure well. No wound complications were reported during his two years follow-up (Fig. 4). Re-Exploration nor revision of the surgery were needed

## 3 Discussion

The coverage of wounds in the head and neck is usually done by using a local skin flap [5]. The disadvantage of using traditional skin flaps is a difficulty shaping them in irregular, large wounds and reaching distal temporal rejoin [1,3].

In 1972 the use of a free omental flap was first done in craniofacial reconstruction.^2^ Many indications were reported, including post tumor resection of squamous cell carcinoma and basal cell carcinoma, and post craniotomy with bone defect [1,4]. However, only one case with a high voltage electrical burn injury was reported, and our case appears to be the first reported flame burn [5].

This case demonstrate the feasibility of minimal invasive procedure of harvest an omental flap laparasopically, and the use of large omental flap to cover the entire scalp of a child skull the probably even latissimus dorsi flap may not provide similar coverage and pliability.

Since then, with more advantages and the use of minimal invasion through the abdomen by laparoscopy instead of open laparotomy, the use of an omental flap is recommended method in scalp and neck reconstruction.

Omental flaps are beneficial because their large size covers wide defects; they are easy to shape in irregular edges over the bony surfaces, and they have high vascularity [3]. Moreover, omental flaps can be either free or pedicle, depending on the site of the flap transfer [6].

Furthermore, with the presence of a long pedicle for micro-anastomoses, the transfers have been easier to perform and have minimal site infection, and no adhesions were reported between the omentum and graft.

Throughout our literature, the gastroepiploic artery was always preserved for the pedicle. In most cases, the right gastroepiploic artery was preserved, in contrast to our case where the left was preserved [5].

Another advantage that makes omental flap a better choice is that minimal or no complications were recorded in both the donor and recipient sites, with a low percentage of flap loss [1]. The only limitation found for performing this reconstructive procedure is abdominal adhesions because of multiple previous surgeries, a laparotomy can be used as an alternative [3].

We think that the use of omental flap is a useful flap and should be considered in the scalp and head and neck reconstruction.

## Conflict of interest

None of the authors has any conflict of interest to declare. The study received no financial support.

## Sources of funding

This research did not receive any specific grant from funding agencies in the public, commercial, or not-for-profit sectors.

## Ethical approval

Case reports are exempted from ethical approval according to policies of King Faisal Specialist Hospital and Research Center.

## Consent

Parental Written informed consent on behalf of the patient has been obtained for publication of this case report.

Copy can be uploaded if requested

## Author contribution

*Noor Allababidi:* study design, data collection, writing the paper. Reviewing and editing the case report.

*Asal Alomaran :* study design, data collection, writing the paper.

*Fuad Hashem:* performed the operation, study concept, reviewing the final manuscript of the case report, final approval.

## Registration of research studies

N/A.

## Guarantor

Dr. Fuad Khaled Hashem.

## Patient perspective

The patient chronic condition of recurrent open wounds for many years because of multiple sharp and irregular surface of the skull caused by multiple drill holes in the outer surface of the skull was treated.

## Patient consent

Parental Written informed consent on behalf of the patient has been obtained for publication of this case report.

Copy can be uploaded if requested

## Provenance and peer review

Not commissioned externally peer reviewed.
